# Association between *CES1* rs2244613 and the pharmacokinetics and safety of dabigatran: Meta-analysis and quantitative trait loci analysis

**DOI:** 10.3389/fcvm.2022.959916

**Published:** 2022-08-04

**Authors:** Haobo Li, Zhu Zhang, Haoyi Weng, Yuting Qiu, Pablo Zubiaur, Yu Zhang, Guohui Fan, Peiran Yang, Anna-Leena Vuorinen, Xianbo Zuo, Zhenguo Zhai, Chen Wang

**Affiliations:** ^1^Department of Pulmonary and Critical Care Medicine, China-Japan Friendship Hospital, Beijing, China; ^2^National Center for Respiratory Medicine, Beijing, China; ^3^Institute of Respiratory Medicine, Chinese Academy of Medical Sciences, Beijing, China; ^4^National Clinical Research Center for Respiratory Diseases, Beijing, China; ^5^Graduate School of Peking Union Medical College, Chinese Academy of Medical Sciences, Beijing, China; ^6^Shenzhen WeGene Clinical Laboratory, Shenzhen, China; ^7^WeGene, Shenzhen Zaozhidao Technology Co., Ltd., Shenzhen, China; ^8^Hunan Provincial Key Lab on Bioinformatics, School of Computer Science and Engineering, Central South University, Changsha, China; ^9^Graduate School of Capital Medical University, Beijing, China; ^10^Clinical Pharmacology Department, Hospital Universitario de La Princesa, Instituto de Investigación Sanitaria, Universidad Autónoma de Madrid, Madrid, Spain; ^11^Department of Clinical Research and Data Management, Center of Respiratory Medicine, China-Japan Friendship Hospital, Beijing, China; ^12^Institute of Basic Medical Sciences, Chinese Academy of Medical Sciences, Peking Union Medical College, Beijing, China; ^13^VTT Technical Research Centre of Finland Ltd., Espoo, Finland; ^14^Department of Dermatology, China-Japan Friendship Hospital, Beijing, China; ^15^Department of Pharmacy, China-Japan Friendship Hospital, Beijing, China

**Keywords:** *CES1*, rs2244613, polymorphism, dabigatran, pharmacokinetics, safety, QTL

## Abstract

**Objective:**

To date, the influence of the carboxylesterase 1 (*CES1*) rs2244613 genotype on the pharmacokinetics (PKs) and safety of dabigatran remains controversial. Hence, a systematic review was performed to study the association between *CES1* rs2244613 genotype and the PKs and safety of dabigatran and *CES1* relative expression.

**Methods:**

In addition to the three English databases (Web of Science, PubMed, and Embase), two Chinese databases (CNKI and Wanfang) were thoroughly revised. The mean differences (MD) and corresponding 95% confidence intervals (CI) were applied to evaluate the differences in PKs between the *CES1* rs2244613 genotype. Odds ratio (OR) was used to study the risk for bleeding events between the *CES1* rs2244613 genotypes. Subsequent expression quantitative trait loci (eQTL) analyses were performed to evaluate genotype-specific expressions in human tissues.

**Results:**

Ten studies (*n* = 2,777) were included. *CES1* rs2244613 G allele carriers exhibited significantly lower dabigatran trough concentrations compared to T allele carriers (MD: −8.00 ng/mL; 95% CI: −15.08 to −0.92; *p* = 0.03). The risk for bleeding events was significantly lower in carriers of the G allele compared to T allele carriers (OR: 0.65; 95% CI: 0.44–0.96; *p* = 0.03). Subsequent eQTL analysis showed significant genome-wide expressions in two human tissues, whole blood (*p* = 5.1 × 10^–10^) and liver (*p* = 6.2 × 10^–43^).

**Conclusion:**

Our meta-analysis indicated a definite relation between the *CES1* rs2244613 genotype and tolerability variations or pharmacokinetic fluctuations. The carriers of T allele showed higher dabigatran concentrations; therefore, they would benefit from a dose reduction.

**Systematic review registration:**

[https://inplasy.com/inplasy-2022-6-0027/], identifier [NPLASY202260027].

## Graphical Abstract

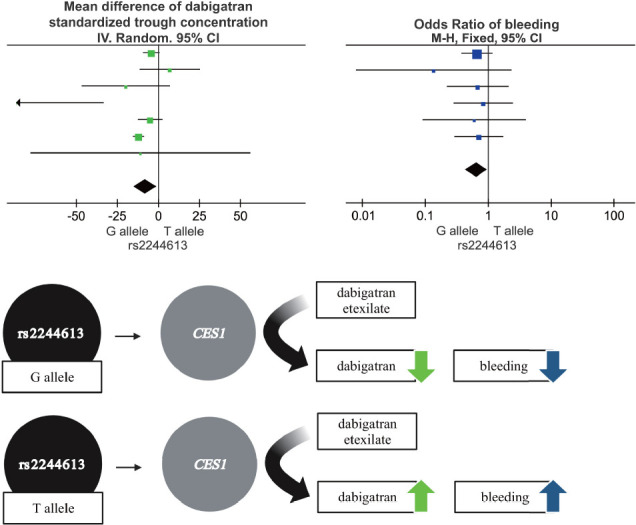

## Introduction

Direct oral anticoagulants (DOACs) are the first alternative to vitamin K antagonists (VKAs) (1). They specifically target a single coagulation protein, including thrombin or coagulation factor Xa. Compared with traditional anticoagulants, the convenience and safety of DOACs is well documented (2). Dabigatran is a representative drug of DOACs widely used to treat atrial fibrillation and pulmonary embolism (3). It is administered as a prodrug–dabigatran etexilate–which is rapidly hydrolyzed into dabigatran, the active moiety, by means of esterases, such as carboxylesterase 1 (*CES1*) and *CES2*. Hepatic *CES1* mainly catalyzes the conversion of the prodrug dabigatran etexilate to dabigatran, while the intestinal *CES2* enzyme plays a compensatory role when *CES1* is inhibited (4). This is the reason why we chose *CES1* as the subject of this study.

*CES1* is a crucial liver enzyme that conduces to the metabolism of drugs containing ester moieties, including dabigatran etexilate or the M1 metabolite (5, 6). As to treatment for atrial fibrillation, *CES1* polymorphism may also affect clopidogrel pharmacological metabolism in the body. Up to 85% of the clopidogrel prodrug entering the body is rapidly hydrolyzed into inactive metabolites under the catalysis of *CES1*, and only 15% of the clopidogrel can exert drug effects. What’s more, *CES1* is related to the development of many other thrombotic diseases like venous thromboembolism through regulating the pharmacokinetics of multiple anticoagulants (7, 8).

Single nucleotide polymorphisms (SNPs) in the *CES1* gene may lead to interindividual differences in dabigatran pharmacokinetics (PKs), which may affect the metabolism and bioavailability of this drug. In addition, although the tolerability of dabigatran is better than that of VKAs, some serious adverse clinical events such as bleeding or thrombosis may occur.

Due to interindividual variability in PKs, bleeding or thrombotic events may occur in patients taking dabigatran. However, the conclusions of the existing studies on the association between the *CES1* SNPs and drug concentration and bleeding risk are controversial due to their small sample sizes (4, 9–11). For instance, *CES1* rs2244613 G allele was related to a reduction in the trough concentration of dabigatran in patients compared to the T allele, and with a reduced risk of bleeding (12, 13). However, Shi et al. (14) observed that this gene locus was unrelated to dabigatran concentration and clinical outcome.

Thence, a systematic review and meta-analysis were conducted with existing studies on the application of dabigatran in atrial fibrillation, cardioembolic stroke, knee arthroplasty, and other diseases. This study explores the relationship between the *CES1* rs2244613 variant and patient’s plasma concentration and bleeding risk and determines its clinical relevance to guide individualized dabigatran prescription further.

## Materials and methods

We performed this study in the light of the preferred reporting items for systematic reviews and meta-analyses (PRISMA) guidelines (Supplementary Table 1) (15). We have registered our detailed protocol for this systematic review on INPLASY (registration number: INPLASY202260027), and it is available in full on inplasy.com^1^.

### Literature search

A structured search of three English databases (Web of Science, PubMed, and Embase) and two Chinese databases (CNKI and Wanfang) was performed on 16 April 2022. The search terms we applied are as follows: (‘novel oral anticoagulant’ or ‘new oral anticoagulant’ or ‘direct oral anticoagulant’ or ‘target-specific oral anticoagulant’ or NOAC or DOAC or TSOAC or dabigatran) and (*CES1* or ‘carboxylesterase 1’ or carboxylesterase-1) and (‘dabigatran concentration’ or bleeding) and (polymorph* or variant* or mutation* or genotyp* or phenotyp* or haplotyp* or SNP or rs2244613).

### Data selection and collection

With duplicate studies removed, two researchers (Li and Qiu) excluded irrelevant studies independently, according to the titles and abstracts and assessed the full-text articles for further inclusion. When inconsistencies occur, a team meeting was held with extra researchers, and a consensus would be finally reached.

In the step of data extraction, a predesigned form to obtain information from the included studies was used, which mainly comprised of basic data (including title, author, date, and sample size) and outcome variables (including means and standard bias for dabigatran plasma levels and the number of bleeding events). Then the means and standard deviations was estimated according to Wan’s method and presented the continuous outcomes in the form of medians and interquartile ranges (16).

### Quality assessment

The Newcastle–Ottawa scale (NOS) tool, which is based on three domains including the selection of exposed and unexposed subjects (0–4 points), comparability of study groups (0–2 points), and outcome assessment (0–3 points), was used to evaluate the quality of the research (17).

### Statistical analysis

The Review Manager software (version 5.3) and STATA software (version 12.0) were used. The MD, OR and 95% CI were used to evaluate the strength of the association. A total of five genetic models were implemented to make an assessment on the association between *CES1* rs2244613 and dabigatran PKs and safety, including: homozygote model (GG vs. TT), heterozygote model (GT vs. TT), dominant model (GG + GT vs. TT), recessive model (GG vs. GT + TT), and allele comparison (G vs. T). The Q and I^2^ statistics were used to evaluate the heterogeneity degree (18). The selection of fixed-effects or random-effects model was based on the degree of heterogeneity (19). *I*^2^ < 50% was considered to low heterogeneity, 50 ≤ *I*^2^ < 75% was considered to moderate heterogeneity and *I*^2^ ≥ 75% was considered to significant heterogeneity. If *I*^2^ < 50% and *p* value > 0.1, the fixed-effects model would be used. If *I*^2^ ≥ 50% or *P* ≤ 0.1, the random-effects model would be used. Multiple populations were enrolled in the present meta-analysis. Therefore, we performed subgroup analysis and evaluated the impact of *CES1* rs2244613 on the dabigatran pharmacokinetics and safety based on diverse ethnicities. To validate the credibility of outcomes in this meta-analysis, a sensitivity analysis was performed to identify potentially influential studies. Furthermore, funnel plot and Egger’s test were applied to detect publication bias (20). The funnel plot depends on whether the points on both sides are symmetric, which indicates a possible publication bias. And Egger’s test depends on the Student’s *t*-test (*p* < 0.05 suggests a publication bias).

### Genotype quantitative trait loci analysis for rs2244613 in human tissues

We assessed the genotype-specific expression of *CES1* in 49 human tissues by *cis*-expression quantitative trait loci (*cis*-eQTL) and splicing quantitative trait loci (sQTL) analysis through the Genotype-Tissue Expression (GTEx) portal^2^ (21). Violin plots of the genotype-specific expression were constructed to visualize normalized gene expressions between three variant genotypes (GG, GT, and TT).

## Results

### Search results and patient characteristics

Fifty four studies were included after the preliminary search, 35 of which remained after removing duplicates. Of 25 removed after full text revision, three were reviews, seven were case reports, six for evaluating other clinical outcomes, and nine for not providing extractable data (Supplementary Figure 1). Finally, ten studies (8, 12, 13, 22–28) involving 2,777 subjects were included: Table 1 summarizes the characteristics of them. The earliest year of included literature is 2013, and the latest year is 2021.

**TABLE 1:** Characteristics of studies included in the systematic review and meta-analysis.

References	Country	Ethnicity	Sample size	Mean age (Years)	Men/Women	BMI (Kg/m^2^)	Dosage regimen	Treatment Indication	NOS
Paré et al. (13)	Canada	Caucasian	1694	71.8	1163/531	29.1	110 mg Bid 150 mg Bid	AF	7
Sychev et al. (8)	Russia	Caucasian	60	62	2/58	35.3	220 mg	Knee replacement	7
Meshcherykov et al. (22)	Russia	Caucasian	72	64.89	35/37	NA	150 mg Bid	AF	7
Xu (23)	China	Asian	113	60.81	68/45	NA	110 mg Bid 150 mg Bid	AF	7
Tomek et al. (24)	Czechia	Caucasian	110	70.2	54/56	NA	NA	Cardioembolic stroke	7
Sychev et al. (12)	Russia	Caucasian	96	75	39/57	29.7	110 mg Bid 150 mg Bid	AF	7
Ji et al. (25)	China	Asian	198	63.3	120/78	23.9	110 mg Bid	AF	7
Lähteenmäki et al. (26)	Finland	Caucasian	340	69.8	178/162	NA	110 mg Bid 150 mg Bid	Multiple diseases	9
Zheng et al. (27)	China	Asian	80	64.5	43/37	23.8	NA	AF	7
Xiang (28)	China	Asian	14	61.5	10/4	24	NA	AF	7

BMI, body mass index; NOS, Newcastle–Ottawa scale; NA, not available; AF, atrial fibrillation; Bid, twice daily; Multiple diseases include vascular disease, stroke/cerebral infarction or atherosclerosis in (pre-)cerebral arteries, atrial Fibrillation, pulmonary embolism, phlebitis and thrombophlebitis, portal vein thrombosis, and other venous embolism and thrombosis.

Seven of the included works analyzed the trough plasma concentration of dabigatran in patients with different genotypes, and nine analyzed the bleeding risk. Six of them were conducted with a Caucasian population and four with Asian populations. All publications were evaluated by NOS and scored above seven points.

### Association between *CES1* rs2244613 and the trough plasma concentration of dabigatran

**FIGURE 1:**
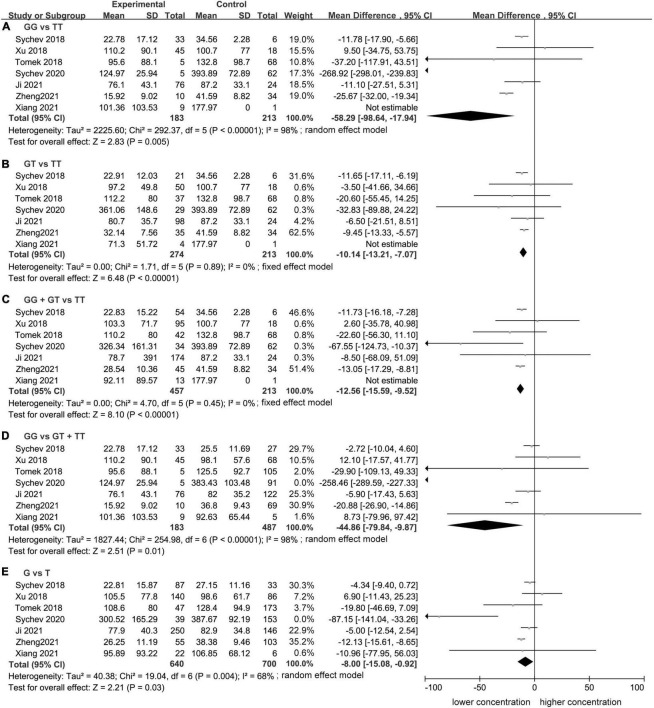
Forest plots comparing dabigatran trough concentrations across rs2244613 genotype polymorphism of the carboxylesterase 1 (*CES1*) gene: **(A)** homozygote model, **(B)** heterozygote model, **(C)** dominant model, **(D)** recessive model, and **(E)** allelic model.

Meta-analysis showed a statistically significant difference between trough plasma concentrations of dabigatran and rs2244613 genotype. In summary, the *CES1* rs2244613 G allele was related to a lower trough plasma concentration of dabigatran when compared with T allele. The following MDs were observed for each model: GG vs. TT, MD = −58.29 ng/mL, 95% CI: −98.64 to −17.94, *P* = 0.005, *I*^2^ = 98%; GT vs. TT: MD = −10.14 ng/mL, 95% CI: −13.21 to −7.07, *P* < 0.00001, *I*^2^ = 0%; GG + GT vs. TT: MD = −12.56 ng/mL, 95% CI: −15.59 to −9.52, *P* < 0.00001, *I*^2^ = 0%; GG vs. GT + TT: MD = −44.86 ng/mL, 95% CI: −79.84 to −9.87, *P* = 0.01, *I*^2^ = 98%; G vs. T: MD = −8.00 ng/mL, 95% CI: −15.08 to −0.92, *P* = 0.03, *I*^2^ = 68% (Figure 1).

**FIGURE 2:**
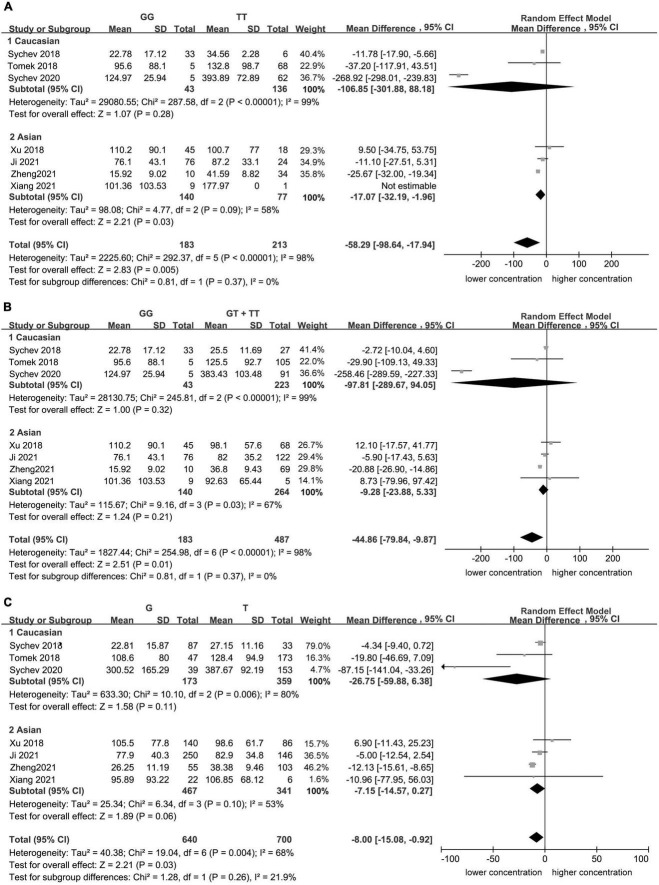
Subgroup analyses for the association between carboxylesterase 1 (*CES1*) rs2244613 polymorphism and the trough plasma concentration of dabigatran: **(A)** homozygote model, **(B)** recessive model, and **(C)** allelic model.

Significant heterogeneity was found for the homozygote model (*I*^2^ = 98%, Figure 1), for the recessive model (*I*^2^ = 98%, Figure 1), and for the allele contrast model (*I*^2^ = 68%, Figure 1). The heterogeneity was lower in Asian population in the homozygote model (*I*^2^ = 58%, Figure 2), recessive model (*I*^2^ = 67%, Figure 2), and allele contrast model (*I*^2^ = 53%, Figure 2).

**FIGURE 3:**
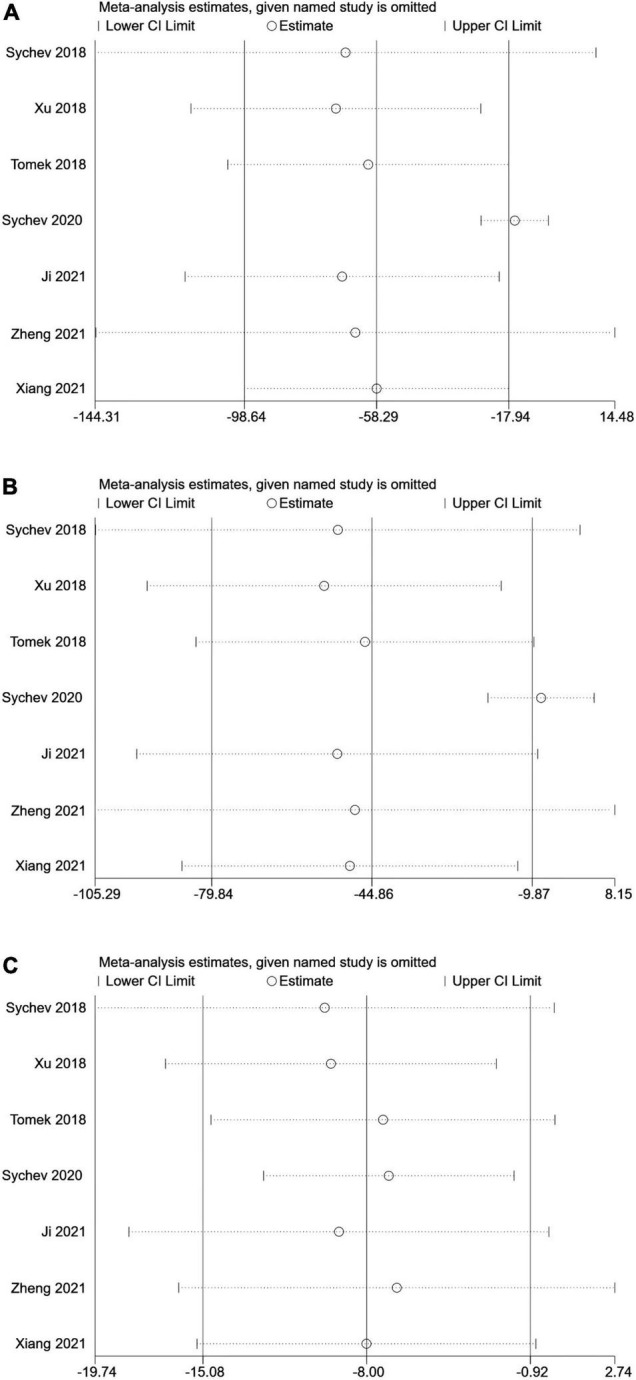
Sensitive analysis of carboxylesterase 1 (*CES1*) rs2244613 polymorphism and the trough plasma concentration of dabigatran: **(A)** homozygote model, **(B)** recessive model, and **(C)** allelic model.

No single study could not influence the overall results qualitatively, indicating robustness and reliability of our results (Figure 3).

**FIGURE 4:**
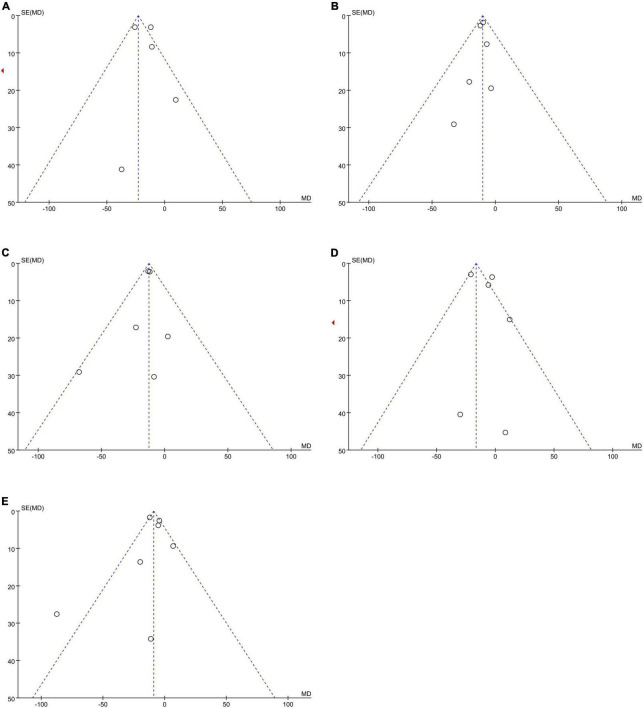
Funnel plots to assess the publication bias related to the dabigatran trough concentrations: **(A)** homozygote model, **(B)** heterozygote model, **(C)** dominant model, **(D)** recessive model, and **(E)** allelic model.

No publication bias was observed, as funnel plots (Figure 4) were relatively symmetrical.

### Association between *CES1* rs2244613 and the risk of bleeding

**FIGURE 5:**
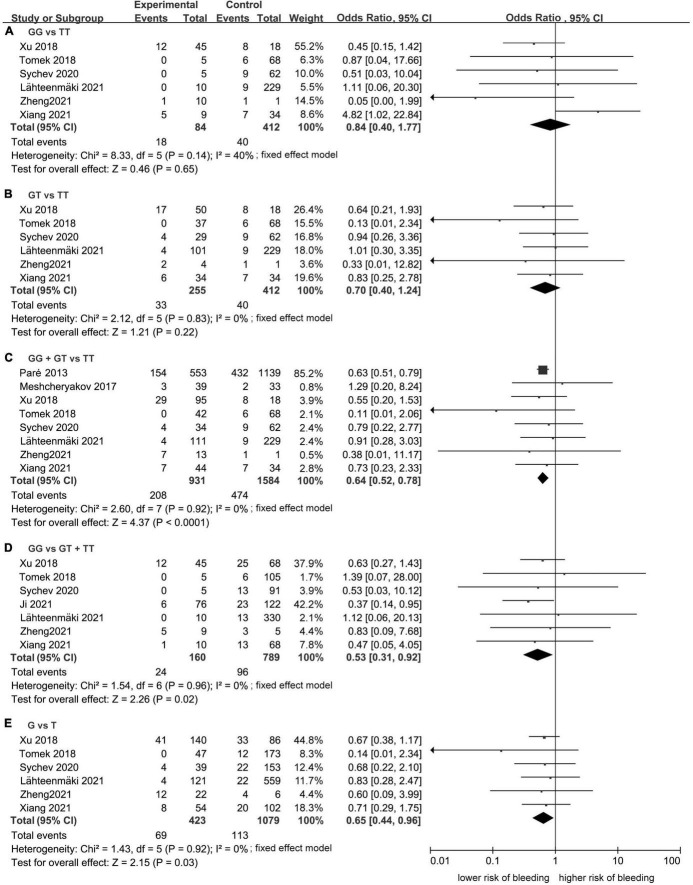
Forest plots comparing the risk of developing any bleeding event across rs2244613 genotype polymorphism of the carboxylesterase 1 (*CES1*) gene: **(A)** homozygote model, **(B)** heterozygote model, **(C**) dominant model, **(D)** recessive model, and **(E)** allelic model.

Meta-analysis showed a statistically significant difference between the risk of developing bleeding and rs2244613 genotype. In summary, the *CES1* rs2244613 G allele was related to a lower risk of developing any bleeding when compared with T allele. The following ORs were observed for each model: GG vs. TT, OR = 0.84, 95% CI: 0.40–1.77, *P* = 0.65, *I*^2^ = 40%; GT vs. TT: OR = 0.70, 95% CI: 0.40–1.24, *P* = 0.22, *I*^2^ = 0%; GG + GT vs. TT: OR = 0.64, 95% CI: 0.52–0.78, *P* < 0.0001, *I*^2^ = 0%; GG vs. GT + TT: OR = 0.53, 95% CI: 0.31–0.92, *P* = 0.02, *I*^2^ = 0%; G vs. T: OR = 0.65, 95% CI: 0.44–0.96, *P* = 0.03, *I*^2^ = 0% (Figure 5).

**FIGURE 6:**
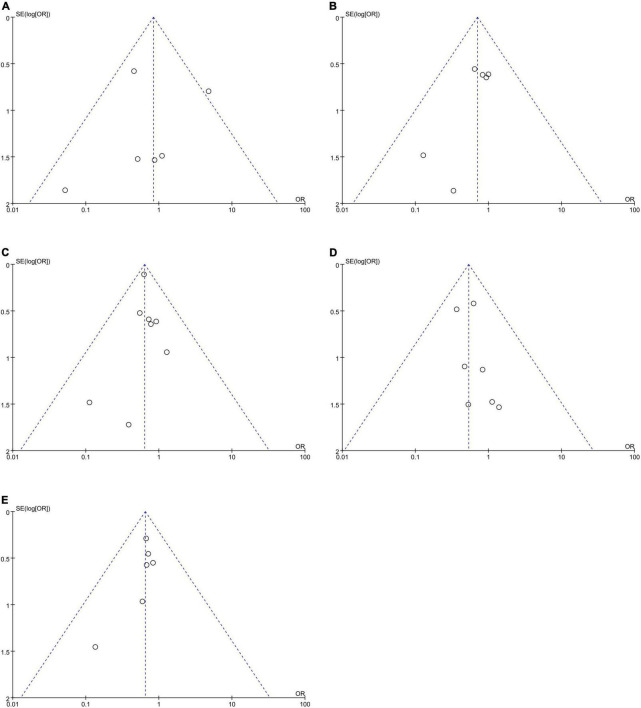
Funnel plots to assess the publication bias related to the risk of bleeding: **(A)** homozygote model, **(B)** heterozygote model, **(C)** dominant model, **(D)** recessive model, and **(E)** allelic model.

No publication bias was observed, as funnel plots (Figure 6) were relatively symmetrical.

### Quantitative trait loci analysis of rs2244613 in human tissues

**FIGURE 7:**
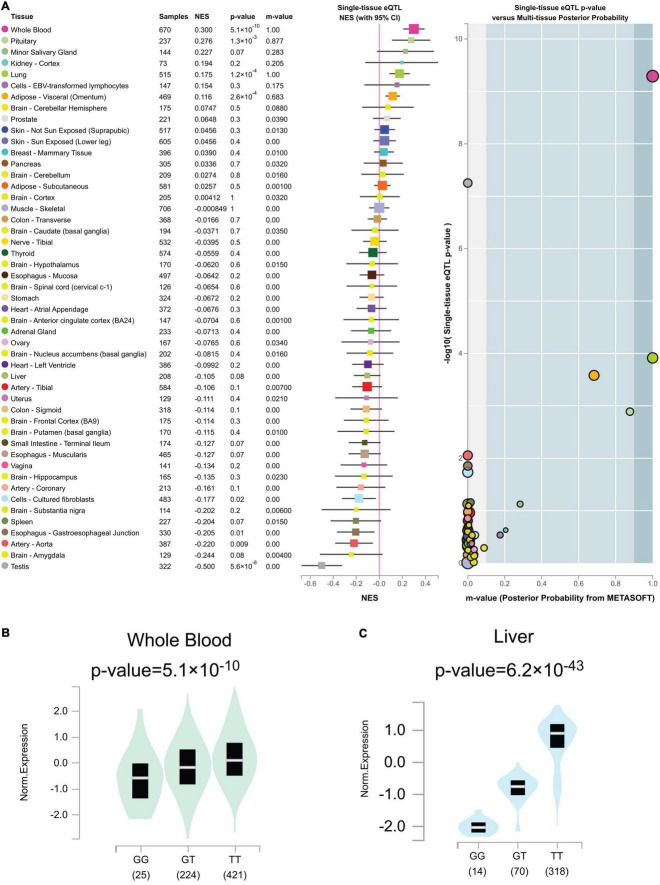
**(A)** Genotype *cis*-expression quantitative trait loci analysis for rs2244613 in 49 human tissues was obtained from the GTE database. **(B)** Violin plots of allele-specific *cis*-eQTLs according to rs2244613 genotypes in whole blood tissue in the Genotype-Tissue Expression (GTEx) dataset. **(C)** Violin plots of allele-specific splicing quantitative trait loci (sQTL) according to rs2244613 genotypes in liver tissue in the GTEx dataset.

Out of the total 49 genotypic *cis*-eQTL results for rs2244613, only one *cis*-eQTLs reached a genome-wide significance threshold in Figure 7A (*p* = 5.1 × 10^–10^ in whole blood tissue). Genome-wide *cis*-eQTLs were upregulated in whole blood tissues in Figure 7B (slope = 0.30). Compared to TT allele patients, the expression of *CES1* was significantly lower in GG. sQTLs showed genome-wide significance in seventeen tissues (*p* < 5 × 10^–8^) in Figure 7C and Supplementary Figure 2. Particularly, finding the *cis*-eQTL and sQTLs genotypes implicated the rs2244613 variant as a transcriptional regulatory factor.

## Discussion

Our study comprehensively explored the application of dabigatran in atrial fibrillation, cardioembolic stroke, and knee replacement, and other diseases to explore the relationship between *CES1* rs2244613 and dabigatran PKs and bleeding risk. 2,777 patients in 10 articles were included. We found that the bleeding risk of patients taking dabigatran with GG and GT genotypes was significantly lower than that of patients with TT genotype; the bleeding risk of patients with GG genotype was remarkably lower than that of patients with GT + TT genotypes. Moreover, the bleeding risk is lower in patients carrying the G allele compare to T allele carriers. Additionally, we consistently observed that the trough concentrations of dabigatran were notably lower in the G compared to the T allele. Therefore, we conclude that *CES1* rs2244613 affects dabigatran plasma concentration and ADR incidence. Moreover, the effect of *CES1* rs2244613 on the trough concentrations of dabigatran varied among ethnicities, which is consistent with previous works (29).

Mammalian CES belong to the α, β-hydrolase-fold protein superfamily, which can be divided into five categories in accordance with the homology of the amino acid sequences (*CES1* – *CES5*). Both *CES1* and *CES2* are mainly involved in the metabolism of human drugs, and *CES1* is mostly found in the human liver (27, 28, 30, 31). Once dabigatran etexilate enters the body, it must be hydrolyzed at two separate sites to form an active thrombin inhibitor. First, in the intestine, the carbamate group is hydrolyzed by *CES2*, while *CES1* hydrolyses the ethyl ester part. After that, it can be converted into dabigatran, which has metabolic activity (5, 14). Then it binds to the specific site of thrombin, inhibiting thrombin activity and preventing fibrin formation, thereby exerting an anticoagulant effect (14).

In fact, apart from *CES1* and *CES2*, there are some other genes encoding enzymes [e.g., UDP-glucuronosyltransferase gene (*UGT*) and cytochrome P450 gene (*CYP*)] and genes encoding transporters [e.g., ATP binding cassette subfamily gene (*ABC*) and solute carriers’ family gene (*SLC*)]. After oral administration, dabigatran binds to plasma proteins and is catalyzed by three UGTs (UGT1A9, UGT2B7, and UGT2B15) to form acyl glucuronic acid isomers, of which UGT2B15 contains the strongest effect. Particularly, dabigatran 1-O-acylglucuronide, a metabolite of dabigatran, exhibited anticoagulant activity comparable to the parent drug (32). In addition, cytochrome P450 (CYP2D6 and CYP3A5) may metabolize dabigatran after CES esterase’s converting dabigatran to the active moiety. Dabigatran is mainly excreted unchanged in urine (85%) and remains in feces (9). Genes encoding transporters are also reported. P-glycoprotein (P-gp) is a classical transporter encoded by the *ABCB1* gene, and dabigatran is one of its substrates. The gene polymorphism of *ABCB1* is considered being related to the pharmacokinetics and drug safety of dabigatran, and has been widely confirmed (33). In addition, SLC family transporters are also involved in the metabolism of dabigatran. For example, studies have shown that the *SLC22A1* mutant haplotype has higher t_max_ and t_1/2_ with dabigatran than heterozygous and wild types, resulting in differences in the pharmacokinetics and safety of dabigatran among users of different genotypes (9).

High interindividual variability in plasma levels of dabigatran was reported, and the coefficient of variation of up to 30% for systemic exposure (34). Genetic variations in drug-metabolizing enzymes, receptors, and transporters have been identified as a major cause of interindividual variability in drug response, potentially leading to differences in responsiveness and adverse reactions to dabigatran therapy among individuals with different genotypes (35). Presently, thousands of SNPs are described in the *CES1* gene, such as rs8192935, rs71647871, and rs2244613 (36). The allele frequency of *CES1* rs2244613 was previously reported to be different in Chinese vs. Caucasian populations, with a G allele prevalence of 61.1% and 15.3–28.3%, respectively. Furthermore, *CES1* rs2244613 G allele was previously associated with reduced trough concentrations and a decreased bleeding risk rather than peak drug concentrations (4, 13, 25, 37). Another study of patients with atrial fibrillation who received oral dabigatran also concluded that the *CES1* SNP rs2244613 was remarkably in association with dabigatran trough concentrations (38). In summary, most conclusions in post researches are consistent with ours, except Xu et al. As a meta-analysis, our study has a large sample size and employs data on dabigatran in a variety of disease populations, only for the drug dabigatran rather than a specific disease, so it has a comparatively high reliability. The reason for the large discrepancy between Xu’s research conclusions and ours may be the limitation of their sample size.

This study still has the following limitations. First, the results of our study indicate that SNPs may directly affect the bleeding risk of dabigatran through an internal mechanism and may indirectly influence the occurrence of adverse events by changing the concentration. The specific mechanisms acquire further basic research. Secondly, this study did not control other factors except genotypes, and the heterogeneity cannot be ignored. Thirdly, the blood concentration of dabigatran used in this study is from a single test rather than the average concentration of multiple tests, which may exist to some extent by chance. Fourthly, we have not analyzed other variants within *CES1* and *CES2*, meta-analysis of other variants will be done in the follow-up.

## Conclusion

In summary, patients carrying at least one *CES1* rs2244613 G allele are associated with decreased dabigatran trough concentrations and lower bleeding risk compared to non-carriers (i.e., with the T/T genotype). This work is of great relevance as it will help eventually in the guidance and individualization of dabigatran prescription.

## Data availability statement

The original contributions presented in this study are included in the article/Supplementary material, further inquiries can be directed to the corresponding authors.

## Author contributions

XZ, ZZhai, and CW had full access to all the data in the study and took responsibility for the content of the manuscript. HW and ZZhang conceived and designed the study. HL and YQ integrated data, analyzed the data, and wrote the manuscript. GF provided methodological support. YZ, PZ, PY, and A-LV participated in editing of the manuscript. All authors were involved in the revision of the manuscript for important intellectual content and approved the final version.

## Footnotes

1
https://inplasy.com/inplasy-2022-6-0027/

2
https://gtexportal.org/home/

## Supplementary material

Supplementary Figure 1 PRISMA flow diagram.

Supplementary Figure 2 Violin plots of allele-specific sQTLs according to rs2244613 genotypes in 17 human tissues in the GTEx dataset.

Supplementary Table 1 PRISMA checklist.

## References

[1] Chan N Sobieraj-Teague M Eikelboom JW. Direct oral anticoagulants: evidence and unresolved issues. *Lancet.* (2020) 396:1767–76. 10.1016/S0140-6736(20)32439-9 33248499

[2] Ruff CT Giugliano RP Braunwald E Hoffman EB Deenadayalu N Ezekowitz MD Comparison of the efficacy and safety of new oral anticoagulants with warfarin in patients with atrial fibrillation: a meta-analysis of randomised trials. *Lancet.* (2014) 383:955–62. 10.1016/S0140-6736(13)62343-0 24315724

[3] Antonijevic NM Zivkovic ID Jovanovic LM Matic DM Kocica MJ Mrdovic IB Dabigatran-metabolism, pharmacologic properties and drug interactions. *Curr Drug Metab.* (2017) 18:622–35. 10.2174/1389200218666170427113504 28460624

[4] Liu Y Yang C Qi W Pei Z Xue W Zhu H The impact of ABCB1 and CES1 polymorphisms on dabigatran pharmacokinetics in healthy chinese subjects. *Pharmgenomics Pers Med.* (2021) 14:477–85. 10.2147/PGPM.S291723 33935512 PMC8081719

[5] Laizure SC Parker RB Herring VL Hu ZY. Identification of carboxylesterase-dependent dabigatran etexilate hydrolysis. *Drug Metab Dispos.* (2014) 42:201–6. 10.1124/dmd.113.054353 24212379 PMC3912543

[6] Shi J Xiao J Wang X Jung SM Bleske BE Markowitz JS Plasma carboxylesterase 1 predicts methylphenidate exposure: a proof-of-concept study using plasma protein biomarker for hepatic drug metabolism. *Clin Pharmacol Ther.* (2022) 111:878–85. 10.1002/cpt.2486 34743324 PMC9249567

[7] Baturina O Andreev D Fedina L Mirzaev K Ivashchenko D Ryzhikova K Influence of clinically significant genes on antiplatelet effect of clopidogrel and clinical outcomes in patients with acute coronary syndrome and atrial fibrillation. *Pharmacology.* (2022) 107:216–26. 10.1159/000521531 35073541

[8] Sychev DA Levanov AN Shelekhova TV Bochkov PO Denisenko NP Ryzhikova KA The impact of ABCB1 (rs1045642 and rs4148738) and CES1 (rs2244613) gene polymorphisms on dabigatran equilibrium peak concentration in patients after total knee arthroplasty. *Pharmacogenomics Pers Med.* (2018) 11:127–37. 10.2147/PGPM.S169277 30100750 PMC6064159

[9] Zubiaur P Saiz-Rodríguez M Ochoa D Navares-Gómez M Mejía G Román M Effect of sex, use of pantoprazole and polymorphisms in SLC22A1, ABCB1, CES1, CYP3A5 and CYP2D6 on the pharmacokinetics and safety of dabigatran. *Adv Ther.* (2020) 37:3537–50. 10.1007/s12325-020-01414-x 32564268

[10] Chin PK Wright DF Zhang M Wallace MC Roberts RL Patterson DM Correlation between trough plasma dabigatran concentrations and estimates of glomerular filtration rate based on creatinine and cystatin C. *Drugs R D.* (2014) 14:113–23. 10.1007/s40268-014-0045-9 24797400 PMC4070467

[11] Roşian A-N Roşian ŞH Kiss B Ştefan MG Trifa AP Ober CD Genotype-phenotype correlation for dabigatran in patients with non-valvular atrial fibrillation (a single centre research). *Hum Vet Med.* (2020) 12: 123–9.

[12] Sychev D Skripka A Ryzhikova K Bochkov P Shevchenko R Krupenin P Effect of CES1 and ABCB1 genotypes on the pharmacokinetics and clinical outcomes of dabigatran etexilate in patients with atrial fibrillation and chronic kidney disease. *Drug Metab Pers Ther.* (2020) 35:20190029. 10.1515/dmpt-2019-0029 32134727

[13] Paré G Eriksson N Lehr T Connolly S Eikelboom J Ezekowitz MD Genetic determinants of dabigatran plasma levels and their relation to bleeding. *Circulation.* (2013) 127:1404–12. 10.1161/CIRCULATIONAHA.112.001233 23467860

[14] Shi J Wang X Nguyen JH Bleske BE Liang Y Liu L Dabigatran etexilate activation is affected by the CES1 genetic polymorphism G143E (rs71647871) and gender. *Biochem Pharmacol.* (2016) 119:76–84. 10.1016/j.bcp.2016.09.003 27614009 PMC5061634

[15] Page MJ McKenzie JE Bossuyt PM Boutron I Hoffmann TC Mulrow CD The PRISMA 2020 statement: an updated guideline for reporting systematic reviews. *BMJ.* (2021) 372:n71. 10.1136/bmj.n71 33782057 PMC8005924

[16] Wan X Wang W Liu J Tong T. Estimating the sample mean and standard deviation from the sample size, median, range and/or interquartile range. *BMC Med Res Methodol.* (2014) 14:135. 10.1186/1471-2288-14-135 25524443 PMC4383202

[17] Wells GA Shea B O’Connell D Peterson J Welch V Losos M *The Newcastle-Ottawa Scale (NOS) for Assessing the Quality of Nonrandomised Studies in Meta-Analyses.* (2002). Available online at: http://www.ohri.ca/programs/clinical_epidemiology/oxford.asp (accessed on 4 April, 2022).

[18] Higgins JP Thompson SG Deeks JJ Altman DG. Measuring inconsistency in meta-analyses. *BMJ.* (2003) 327:557–60. 10.1136/bmj.327.7414.557 12958120 PMC192859

[19] Mantel N Haenszel W. Statistical aspects of the analysis of data from retrospective studies of disease. *J Natl Cancer Inst.* (1959) 22:719–48. 13655060

[20] Egger M Davey Smith G Schneider M Minder C. Bias in meta-analysis detected by a simple, graphical test. *BMJ.* (1997) 315:629–34. 10.1136/bmj.315.7109.629 9310563 PMC2127453

[21] GTEx Consortium. Human genomics. The genotype-tissue expression (GTEx) pilot analysis: multitissue gene regulation in humans. *Science.* (2015) 348:648–60. 25954001 10.1126/science.1262110 PMC4547484

[22] Meshcheryakov YV Chertovskikh YV Sychev DA. The pharmacogenetics of the new oral anticoagulant dabigatran - the role of rs2244613 CES1 polymorphism in the adverse reactions development. *Pharmacogenet Pharmacogenomics.* (2017) 2:18–9.

[23] Xu L. *Study on TDM Monitoring, Hemorrhage Risk and Thrombosis Risk of Dabigatran Etexilate in NVAF Patients for Atrial Fibrillation Ablation in China (Chinese).* Master’s thesis. Shanghai Jiao Tong University, Shanghai (2018).

[24] Tomek A Jansky P Olserova A Cederquist L. The correlation of through plasmatic concentration of dabigatran and CES1 genotype with major bleeding in stroke patients. *Stroke.* (2018) 49:ATM110. 10.1161/str.49.suppl_1.TMP110

[25] Ji Q Zhang C Xu Q Wang Z Li X Lv Q. The impact of ABCB1 and CES1 polymorphisms on dabigatran pharmacokinetics and pharmacodynamics in patients with atrial fibrillation. *Br J Clin Pharmacol.* (2021) 87:2247–55. 10.1111/bcp.14646 33179295

[26] Lähteenmäki J Vuorinen AL Pajula J Harno K Lehto M Niemi M Pharmacogenetics of bleeding and thromboembolic events in direct oral anticoagulant users. *Clin Pharmacol Ther.* (2021) 110:768–76. 10.1002/cpt.2316 34043814

[27] Zheng X Zhang L Zhang J. Effects of ABCB1 and CES1 genetic polymorphisms on the anticoagulant efficacy of dabigatran etexilate in patients with non-valvular atrial fibrillation (Chinese). *Chin J Clin Lab Sci.* (2021) 39:903–8.

[28] Xiang J. *Correlation Study of ABCB1 and CES1 Gene Polymorphisms with Rivaroxaban/Dabigatran Plasma Concentration and Drug Safety in Patients with Atrial Fibrillation (Chinese).* Master’s thesis, ChongQing Medical University, Chongqing (2021).

[29] Sychev DA Abdullaev SP Mirzaev KB Ryzhikova KA Shuyev GN Sozaeva ZA Genetic determinants of dabigatran safety (CES1 gene rs2244613 polymorphism) in the Russian population: multi-ethnic analysis. *Mol Biol Rep.* (2019) 46:2761–9. 10.1007/s11033-019-04722-w 30850966

[30] Hosokawa M Furihata T Yaginuma Y Yamamoto N Koyano N Fujii A Genomic structure and transcriptional regulation of the rat, mouse, and human carboxylesterase genes. *Drug Metab Rev.* (2007) 39:1–15. 10.1080/03602530600952164 17364878

[31] Laizure SC Herring V Hu Z Witbrodt K Parker RB. The role of human carboxylesterases in drug metabolism: have we overlooked their importance? *Pharmacotherapy.* (2013) 33:210–22. 10.1002/phar.1194 23386599 PMC4572478

[32] Ebner T Wagner K Wienen W. Dabigatran acylglucuronide, the major human metabolite of dabigatran: in vitro formation, stability, and pharmacological activity. *Drug Metab Dispos.* (2010) 38:1567–75. 10.1124/dmd.110.033696 20551237

[33] Yamazaki S Evers R De Zwart L. Physiologically-based pharmacokinetic modeling to evaluate in vitro-to-in vivo extrapolation for intestinal P-glycoprotein inhibition. *CPT Pharmacometrics Syst Pharmacol.* (2022) 11:55–67. 10.1002/psp4.12733 34668334 PMC8752109

[34] Stangier J Rathgen K Stähle H Gansser D Roth W. The pharmacokinetics, pharmacodynamics and tolerability of dabigatran etexilate, a new oral direct thrombin inhibitor, in healthy male subjects. *Br J Clin Pharmacol.* (2007) 64:292–303. 10.1111/j.1365-2125.2007.02899.x 17506785 PMC2000643

[35] Chen F Zhang B Parker RB Laizure SC. Clinical implications of genetic variation in carboxylesterase drug metabolism. *Expert Opin Drug Metab Toxicol.* (2018) 14:131–42. 10.1080/17425255.2018.1420164 29264996

[36] Raymond J Imbert L Cousin T Duflot T Varin R Wils J Pharmacogenetics of direct oral anticoagulants: a systematic review. *J Pers Med.* (2021) 11:37. 10.3390/jpm11010037 33440670 PMC7826504

[37] Hamzic S Kummer D Milesi S Mueller D Joerger M Aebi S Novel genetic variants in carboxylesterase 1 predict severe early-onset capecitabine-related toxicity. *Clin Pharmacol Ther.* (2017) 102:796–804. 10.1002/cpt.641 28139840

[38] Dimatteo C D’Andrea G Vecchione G Paoletti O Cappucci F Tiscia GL Pharmacogenetics of dabigatran etexilate interindividual variability. *Thromb Res.* (2016) 144:1–5. 10.1016/j.thromres.2016.05.025 27261537

