# Supplementary figures and images for: Association between CES1 rs2244613 and the pharmacokinetics and safety of dabigatran: Meta-analysis and quantitative trait loci analysis
Source: Front Cardiovasc Med. 2022 Aug 4;9:959916. doi: 10.3389/fcvm.2022.959916 (PMC9386138; doi:10.3389/fcvm.2022.959916)

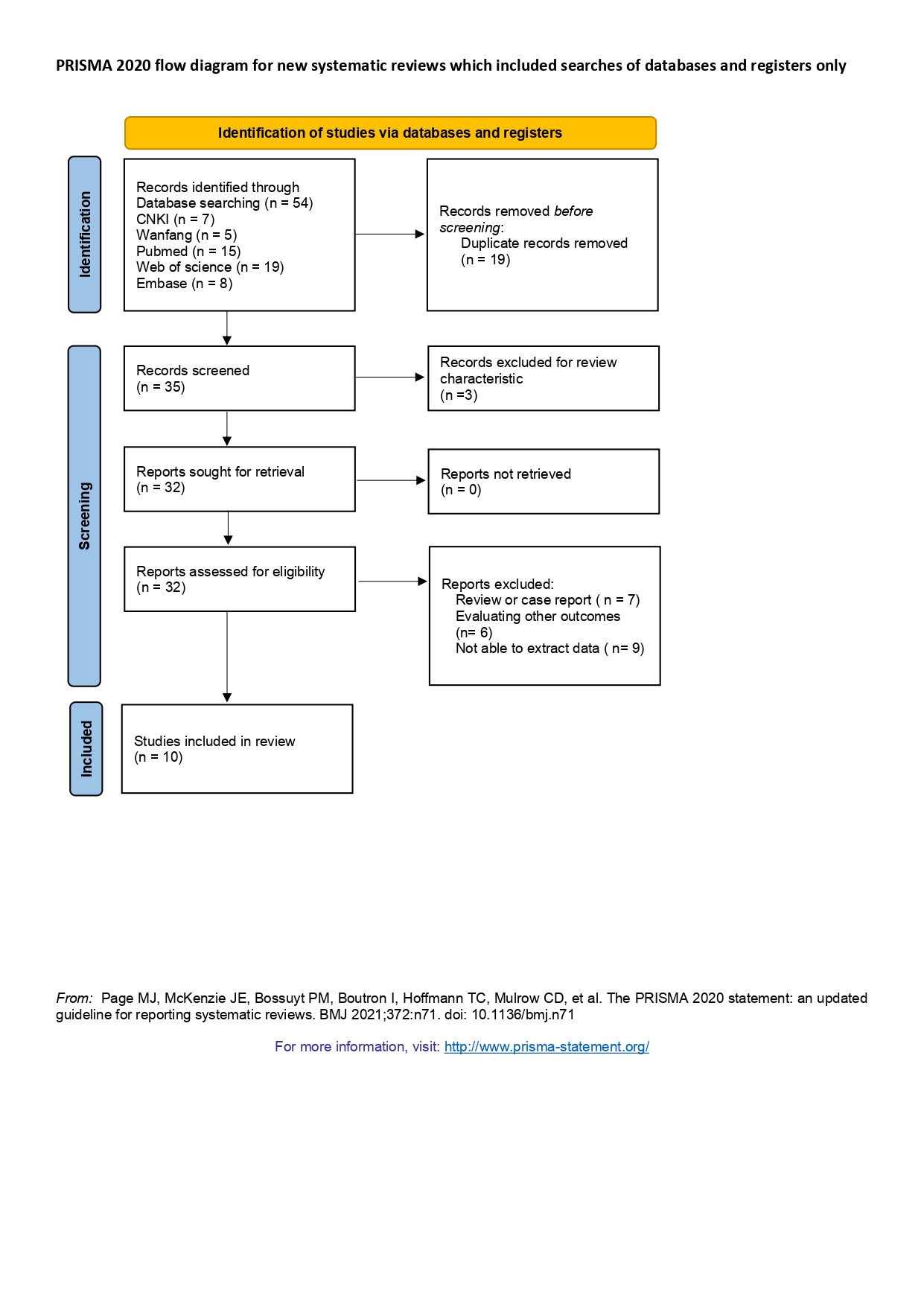

Supplement: Supplementary Figure 1 — PRISMA flow diagram. [file Image_1.jpg]

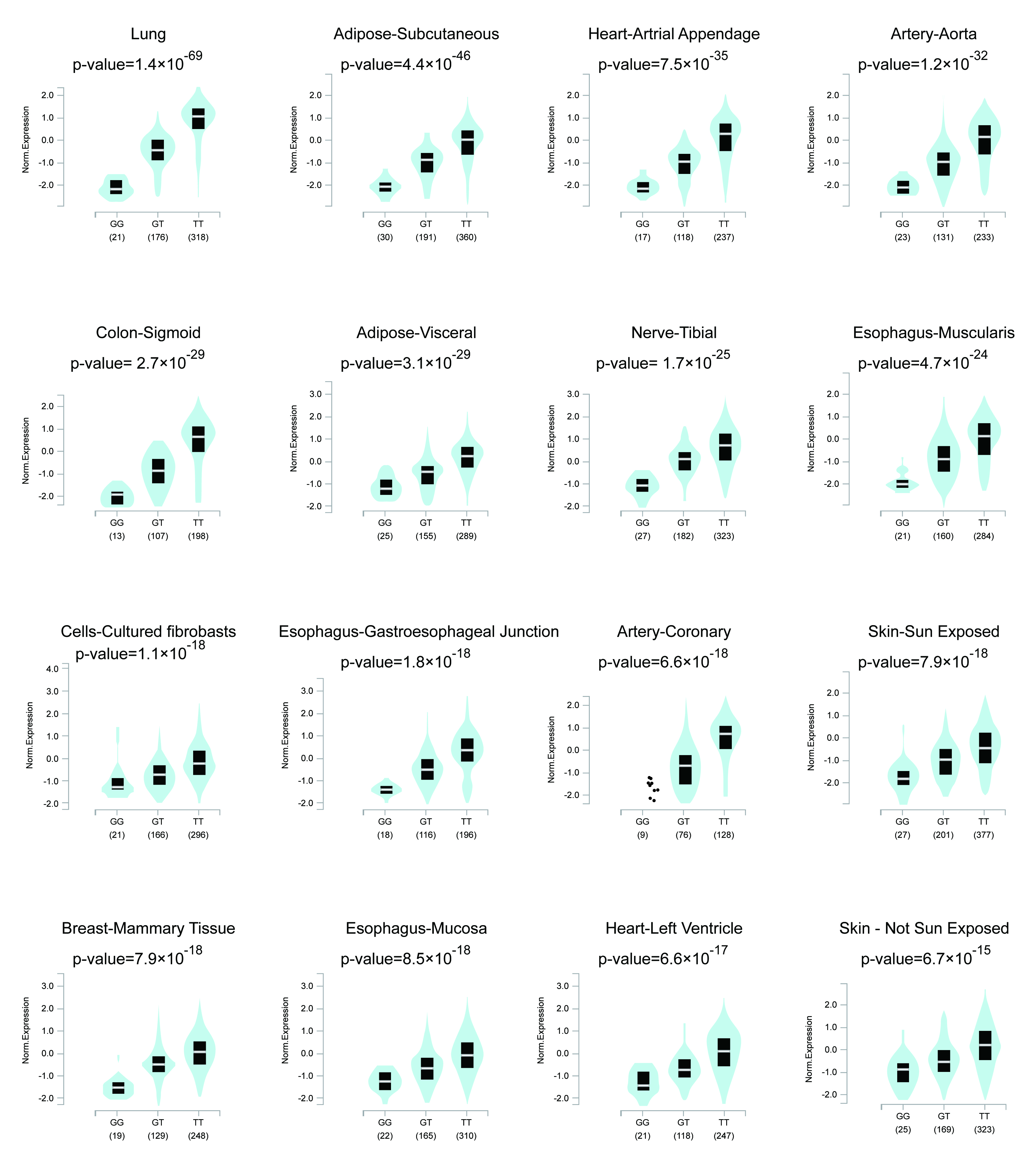

Supplement: Supplementary Figure 2 — Violin plots of allele-specific sQTLs according to rs2244613 genotypes in 17 human tissues in the GTEx dataset. [file Image_2.jpg]
